# The first report on normal sagittal spinopelvic alignment of Japanese population using EOS imaging

**DOI:** 10.1186/1748-7161-10-S1-O35

**Published:** 2015-01-19

**Authors:** Kazuhiro Hasegawa, Masashi Okamoto, Keiji Ishii, Haruka Shimoda, Takao Homma

**Affiliations:** 1 Niigata Spine Surgery Center, Niigata, Japan

## Purpose

Normal spinal alignment in standing position can be precisely measured by using EOS imaging which also allows 3-D simulation of the spine. Although sagittal alignment of the spine has been investigated using a conventional x-ray, the data is yet sparse. The purpose of this study is to clarify the alignment of the spine in normal Japanese population as accurate as possible.

## Methods

One-hundred thirty six volunteers (male:34, female:102, mean BMI=21.1) who have no history of spinal diseases were enrolled. The average age was 39.5 years (20 ~ 69). Spino-pelvic alignments (T1/T12 kyphosis, Lumbar Lordosis [LL], Sacral Slope [SS], Pelvic tilt [PT], Pelvic Incidence [PI], T9-hip axis sagittal angle [T9ang], and sagittal vertical axis [SVA]) were measured using the EOS imaging system which was firstly installed in Japan.

The subjects were divided into 5 generations: 20's (n=30), 30's (n=43), 40's (n=35), 50's (n=20), and 60's (n=8). Then, the data were compared among the generations.

## Results

Average±SD of T1/T12 kyphosis, LL, SS, PT, PI, T9ang, and SVA were 41.7±9.6 (deg), 55.4±1 1.3 (deg), 40.7±8.7 (deg), 11.7±7.8 (deg), 52.5±11.9 (deg), 9.2±2.9 (deg), and 0.21±2.31 (cm), respectively. The average of PI in each generation were 48.6 in 20's, 52.3 in 30's, 54.5 in 40's, 53.7 in 50's, and 57.3 in 60's with no significant difference. T1/T12 kyphosis, PT, and SVA showed a tendency of greater value with higher generation. On the other hand, there was no significant change in LL, SS, PI, and T9ang.

## Conclusion

Sagittal alignment of normal Japanese population was almost comparable to the previous reports (Vialle 2005, Roussouly 2006, Mac-Thiong 2008, Lafage 2008, Kanemura 2009). The results in present study support a hypothesis that PI is an inherent value to the individual, suggesting that the other parameters such as thoracolumbar curves and orientation of the pelvis control the sagittal balance of the spine.

